# The association between polluted fuel use and self-reported insomnia symptoms among middle-aged and elderly Indian adults: a cross-sectional study based on LASI, wave 1

**DOI:** 10.1186/s12889-023-16836-9

**Published:** 2023-10-09

**Authors:** Siqi Leng, Yuming Jin, Michael V. Vitiello, Ye Zhang, Rong Ren, Lin Lu, Jie Shi, Xiangdong Tang

**Affiliations:** 1 Sleep Medicine Center, Department of Urology, Mental Health Center, Department of Respiratory and Critical Care Medicine, West China Hospital, Sichuan University, Dian Xin Nan Jie 28#, Chengdu, 610041 China; 2 Department of Psychiatry and Behavioral Sciences, University of Washington School of Medicine, Seattle, WA 98195 USA; 3 National Institute on Drug Dependence and Beijing Key Laboratory of Drug Dependence Research, Peking University, Beijing, 100191 China

**Keywords:** Polluted fuel use, Insomnia, India, Indoor air pollution

## Abstract

**Background:**

Insomnia predisposes the aging population to reduced quality of life and poor mental and physical health. Evidence of the association between polluted fuel use and insomnia symptoms is limited and is non-existent for the Indian population. Our study aimed to explore the link between polluted fuel use and insomnia symptoms in middle-aged and older (≥ 45 years) Indian populations.

**Methods:**

We utilized data from nationally representative Longitudinal Aging Study in India (LASI) Wave 1. Participants with complete information on fuel use, insomnia symptoms, and covariates were included. Insomnia symptoms were indicated by the presence of at least one of three symptoms: difficulty in initiating sleep (DIS), difficulty in maintaining sleep (DMS), or early morning awakening (EMA), ≥ 5 times/week. Survey-weighted multivariable logistic regression analyses were conducted to evaluate the association between polluted fuel use and insomnia symptoms. We also assessed the interaction of association in subgroups of age, gender, BMI, drinking, and smoking status.

**Results:**

Sixty thousand five hundred fifteen participants met the eligibility criteria. Twenty-eight thousand two hundred thirty-six (weighted percentage 48.04%) used polluted fuel and 5461 (weighted percentage 9.90%) reported insomnia symptoms. After full adjustment, polluted fuel use was associated with insomnia symptoms (OR 1.16; 95%CI 1.08–1.24) and was linked with DIS, DMS, and EMA (OR 1.14; 95%CI 1.05–1.24, OR 1.12; 95%CI 1.03–1.22, and OR 1.15; 95%CI 1.06–1.25, respectively). No significant interactions for polluted fuel use and insomnia symptoms were observed for analyses stratified by age, sex, BMI, drinking, or smoking.

**Conclusions:**

Polluted fuel use was positively related to insomnia symptoms among middle-aged and older Indians. Suggestions are offered within this article for further studies to confirm our results, to explore underlying mechanisms, and to inform intervention strategies.

## Background

Approximately 2.4 billion people worldwide are continuously dependent on polluted fuels (such as kerosene, charcoal, coal, crop residue, wood, and dung) for domestic purposes, most of them are rural residents in low- and middle-income countries (LMICs) including India [1]. 59% of the Indian population lacks access to clean cooking [2]. The incomplete combustion of polluted fuels generates a range of health-damaging pollutants, including fine particulate matter smaller than 2.5 μm (PM_2.5_), carbon monoxide (CO), black carbon (BC), becoming the primary source of household air pollution (HAP) [3]. Currently, HAP relating to polluted fuel is considered to be the main contributor to particulate exposure in LMICs [4]. These small particulates can penetrate the lungs and enter the bloodstream, causing health hazards via mucociliary dysfunction, impaired immune response, and decreased oxygen-carrying capacity of blood [4]. Several recent studies have indicated that polluted fuel exposure is correlated with and a risk factor for stroke, ischemic heart disease, respiratory infections, chronic obstructive pulmonary disease (COPD), and respiratory tract cancer [5, 6]. Moreover, HAP sourced from polluted fuel is the tenth leading risk factor for attributable disability-adjusted life years (DALYs) [7]. According to World Health Organization, estimates of 3.2 million premature deaths per year are attributable to the HAP generated by the use of polluted fuels, with about 0.8 million Indians dying prematurely of HAP in 2020 [8].

Insomnia is clinically diagnosed based on the self-report of experiencing difficulties in falling and staying asleep or waking up earlier than intended associated with daytime functional deficits given adequate opportunity to sleep. This pattern must persist for at least 3 days per week and for at least three months [9]. Insomnia has grown into a global epidemic, affecting approximately 10% to 20% of the general population, of which about half experience insomnia chronically [10]. In India, insomnia is prevalent in approximately 15% of middle-aged and older populations [11], and it often occurs comorbidly with mental distress, cognitive deficits, cardiovascular disorders, and metabolic syndrome and is associated with direct, indirect, and intangible medical and occupational costs [12, 13].

Contaminated air can impair essential cellular functions and lead to aberrant accelerated epigenetic clocks, oxidative stress, and inflammation, impairing central nervous system function, thereby contributing to insomnia [14]. The detrimental effects of ambient air pollution (AAP) on insomnia are evident from existing studies. Studies in China have reported links between long-term [15–18] and short-term [19] AAP and sleep disorders. Positive associations have also been observed of long-term AAP with overall sleep health among participants from the UK Biobank [20]. And researchers have linked maternal PM_2.5_ exposure with sleep disruption among Mexican preschoolers [21]. However, evidence of the association between HAP and sleep health remains sparse. Potential relationships between polluted fuel use and impaired sleep quality have been suggested by several cross-sectional [22–24] and cohort [25, 26] studies in the Chinese population. Those studies solely focus on the Chinese population, with their ability to extrapolate to other populations restricted. Nevertheless, similar relationships remain unexplored in the Indian population, despite the excessive dependence on polluted fuels in Indian households. Our study was conducted to assess the links of different fuel types with insomnia symptoms, employing the baseline data from the Longitudinal Ageing Study in India (LASI). We hypothesized that individuals relying on polluted fuels, in comparison to those utilizing clean fuels, would have higher prevalence rates of insomnia symptoms.

## Methods

### Data

**Fig. 1:**
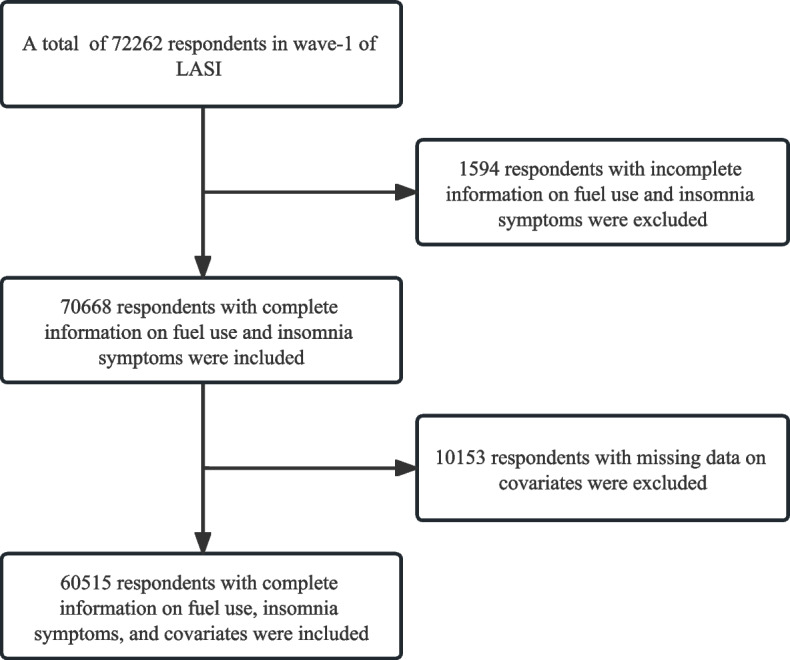
Sample selection for the study

Data for the study were drawn from the first wave of LASI, collected from 2017 to 2018. It is a national survey of 72,262 individuals aged ≥ 45 years and their spouses (irrespective of age), across all states and union territories of India excluding Sikkim [27]. LASI mainly focused on the health status and socioeconomic determinants and consequences of population aging [28]. LASI survey adopted a multi-stage stratified area probability clustering sampling design to reach the final sampling units. We utilized the Harmonized LASI data to investigate the individual, household, and community information of the same observation unit. The detailed study methodology and microdata have been explained in the Harmonized LASI report [29]. Ethical approvals for LASI were obtained from the Indian Council of Medical Research (ICMR) and all collaborating institutions, including the International Institute for Population Sciences (IIPS), Harvard T.H. Chan School of Public Health (HSPH), University of Southern California (USC), ICMR-National AIDS Research Institute (NARI), and Regional Geriatric Centres (RGCs). Informed consent was obtained from all survey participants prior to the first wave of panel surveys. After removing participants with missing data on fuel use, insomnia symptoms, and covariates the total sample size for this study was 60,515 (see Fig. 1).

### Fuel use

Detailed data about fuel types for cooking and non-cooking purposes (e.g., boiling water, bathing, lighting, etc.) were collected. Clean fuels included Liquefied Petroleum Gas (LPG), biogas, and electricity; polluted fuels consisted of kerosene, charcoal, coal, crop residue, wood/shrub, and dung cake.

### Insomnia symptoms

Insomnia symptoms were identified by the presence of at least one of the symptoms: 1) difficulty in falling asleep; 2) difficulty in staying asleep or getting back to sleep after awakening; 3) waking up earlier than intended. The frequency of insomnia symptoms was also recorded, as never or rarely (0–2 times per week), occasionally (3–4 times per week), and frequently (5 or more times per week). A participant was classified as having insomnia symptoms based on reporting at least one of the three symptoms occurring frequently (5 or more times a week). Correspondently, those reporting “experiencing difficulty falling asleep” frequently were classified as difficulty in initiating sleep (DIS), those reporting “experiencing difficulty staying asleep or getting back to sleep after awakening” frequently were classified as difficulty in maintaining sleep (DMS), and those reporting “waking up earlier than intended” frequently were classified as early morning awakening (EMA).

### Covariates

Covariates were chosen based on existing studies and LASI data availability. Demographic and socioeconomic covariates (age categories, sex, level of education, working status, marital status, living arrangement, economic status, religion, and caste) were included. Religion has been recognized as a social determinant of sleep. It may potentially impact sleep by modulating psychological distress, substance use, stress exposure, and allostatic load [30]. Economic status was trichotomized into - low, middle, and high - based on annual per capita household consumption [31]. Annual per capita household consumption was calculated by dividing the total household consumption by the number of households. The total household consumption was the sum of expenditures on food, household utilities, fees, durable goods, education, transit, remittances, discretionary spending, and outpatient and inpatient health care in the previous year. Caste was a hereditary social class of traditional Hindu society, distinguished by relative degrees of wealth, inherited rank or privilege, occupation, and ritual purity or pollution [32]. Caste was classified into scheduled caste, scheduled tribal, other backward class, and none of the above in our study.

Other sources of indoor pollution were assessed, including exposure to other indoor pollutants such as incense sticks, mosquito coils, liquid vaporizers/mosquito repellents/mats, fast cards/sticks/cakes, or exposure to passive smoking indoors, and the responses were coded as “no” or “yes”. Five indicators of poor housing quality were chosen based on previous studies, as a low socioeconomic status (SES) marker [33], including: a) housing construction material (semi-pucca/ kutcha was coded as 1, pucca was coded as 0); b) sanitary facility (poor sanitation was coded as 1, improved sanitation was coded as 0); c) source of water (poor drinking water source was coded as 1, improved drinking water source was coded as 0); d) electricity (without electricity was coded as 1, with electricity was coded as 0) and e) crowding (without separate bedroom was coded as 1, with separate bedroom was coded as 0). The total number of indicators of poor housing quality was calculated and classified as “0/1/ ≥ 2” indicators. Body mass index (BMI) was categorized as < 18.5, 18.5–23, 23–25, and ≥ 25 kg/m^2^, according to the world health organization (WHO) criteria for Asian population [34]. Vigorous physical activity was defined as participation in running or jogging, swimming, going to a health center/gym, cycling, digging with a spade or shovel, heavy lifting, chopping, farm work, fast bicycling, and cycling with loads and was classified by weekly frequency. The number of chronic diseases was classified into “0/1/2/3+” groups according to the sum of self-reported diseases. Chronic diseases included hypertension, diabetes, tumor, lung disease, chronic heart disease, stroke, arthritis, mental disease, Alzheimer’s disease, hypercholesterolemia, asthma, congestive heart failure, heart attack, abnormal heart rate, osteoporosis, abnormal thyroid function, digestive disease, skin disease, kidney stone, presbyopia, cataract, glaucoma, myopia, hyperopia, tooth decay, and periodontal disease. Drinking and smoking status was recorded as “never”, “ever” and “current”. Depression was screened through the 10-item Center for Epidemiologic Studies Depression Scale (CES-D-10), a short version of CES-D-20 [35]. The 10 items of CES-D-10 analyzed seven negative items and three positive items. Each negative item had a score ranging between 0 to 3, and the scores of positive items were reversed. The sum of the scores for each item ranged from 0 to 30 and scoring more than ten was classified as depression in our study, which was verified to have good sensitivity in the previous study [36].

### Statistical analysis

Considering the complex sampling design employed in LASI, all analyses conducted in this study incorporated sample weights. Descriptive statistics stratified by fuel use were calculated. Categorical variables were reported as weighted percentages, and continuous variables as weighted means, accompanied by corresponding 95% confidence intervals (CIs). Differences between the groups were evaluated by the Kruskal Wallis H test for continuous variables and chi-squared tests for categorical variables. Survey-weighted multivariate logistic regression was employed adjusting for different sets of covariates to estimate the links of fuel use with insomnia symptoms and each of the three kinds of symptoms, including DIS, DMS, and EMA. No covariates were adjusted in unadjusted model. Model 1 adjusted for demographic and socioeconomic covariates, including age categories, sex, level of education, work status, marital status, living arrangement, place of residence, economic status, religion, and caste. Model 2 adjusted for the covariates in model 1 and added biologic covariates, such as body mass index (BMI), vigorous physical activity, number of chronic diseases, drinking status, smoking status, and depression. Model 3 adjusted for the covariates in model 2 and added environmental covariates, involving other indoor pollution and indicators of poor housing quality. We employed the likelihood ratio test and Hosmer-Lemeshow test to evaluate the goodness of fit of the fully adjusted model 3.

We also assessed the interaction of association between fuel use and insomnia symptoms in prespecified subgroups including age categories, sexes, BMIs, drinking status, and smoking status. Stratified logistic regression models were employed to perform subgroup analyses, and a log-likelihood ratio test was used to calculate the p for interaction. Data were analyzed via the statistical packages R (The R Foundation; http://www.R-project.org) and Empower (http://www.empowerstats.com). Two-sided *p*-values of less than 0.05 were considered statistically significant.

## Results

### Baseline characteristics

The descriptive statistics of eligible participants, stratified by fuel use, are summarized in Table 1. Among the 60,515 participants, the weighted mean age was 57.58 years (95%CI 46.98–68.18 years). Twenty-eight thousand three hundred twenty-six (weight percentage 48.04%, 95%CI 47.65%-48.44%) participants were using polluted fuels. Participants using polluted fuels were more inclined to be older, lower education level, employed, unmarried, living alone, residing in rural regions, lower economic status, Hindu, the scheduled castes and scheduled tribes, BMI < 23 kg/m^2^, regular physical activity, current smoker, and drinker, lower exposure to other indoor pollution, and poorer housing quality. Overall, the weighted prevalence of insomnia symptoms was 9.90% (95%CI 9.66%-10.14%). A higher weighted prevalence of insomnia symptoms was reported among participants who relied on polluted fuels compared with participants who relied on clean fuels (weighted percentage 10.86% vs 9.01%, *p* < 0.001).

**Table 1:** Weighted baseline characteristics of participants

Characteristics	No.	Overall	Fuel use		
			Clean (*n* = 32,279)	Polluted (*n* = 28,236)	*p*-value
Age, years	60,515	57.58 (10.60)	57.32 (10.61)	57.87 (10.58)	< 0.001^*^
Sex					0.343^†^
Male	25,497	50.52 (50.12–50.91)	50.33 (49.78–50.88)	50.72 (50.14–51.29)	
Female	35,018	49.48 (49.09–49.88)	49.67 (49.12–50.22)	49.28 (48.71–49.86)	
Level of education					< 0.001^†^
No schooling	27,804	51.93 (51.53–52.33)	38.52 (37.98–39.05)	66.44 (65.89–66.98)	
Less than 5 years complete	21,251	29.54 (29.18–29.91)	32.32 (31.80–32.84)	26.54 (26.03–27.05)	
5–9 years complete	8177	13.03 (12.76–13.30)	19.63 (19.20–20.08)	5.89 (5.62–6.16)	
10 or more years complete	3283	5.50 (5.32–5.69)	9.53 (9.21–9.86)	1.14 (1.02–1.27)	
Working status					< 0.001^†^
Currently unemployed	30,396	43.89 (43.50–44.29)	48.05 (47.50–48.60)	39.39 (38.83–39.95)	
Currently employed	30,119	56.11 (55.71–56.50)	51.95 (51.40–52.50)	60.61 (60.05–61.17)	
Insomnia symptoms					< 0.001^†^
No	55,054	90.10 (89.86–90.34)	90.99 (90.67–91.31)	89.14 (88.77–89.49)	
Yes	5461	9.90 (9.66–10.14)	9.01 (8.69–9.33)	10.86 (10.51–11.23)	
Other indoor pollution					< 0.001^†^
No	6777	8.85 (8.63–9.08)	8.15 (7.85–8.46)	9.61 (9.27–9.95)	
Yes	53,738	91.15 (90.92–91.37)	91.85 (91.54–92.15)	90.39 (90.05–90.73)	
Indicators of poor housing quality					< 0.001^†^
0	22,691	34.98 (34.60–35.36)	50.77 (50.22–51.32)	17.91 (17.47–18.36)	
1	20,268	30.37 (30.00–30.73)	30.25 (29.75–30.76)	30.49 (29.96–31.02)	
≥ 2	17,556	34.65 (34.27–35.03)	18.98 (18.55–19.41)	51.60 (51.03–52.18)	
BMI, kg/m2					< 0.001^†^
< 18.5	10,839	20.13 (19.81–20.45)	11.06 (10.72–11.41)	29.93 (29.41–30.46)	
18.5–23	22,449	38.00 (37.61–38.39)	32.85 (32.33–33.37)	43.57 (43.00–44.14)	
23–25	9164	14.67 (14.39–14.95)	17.19 (16.77–17.61)	11.95 (11.58–12.32)	
≥ 25	18,063	27.21 (26.85–27.56)	38.90 (38.37–39.44)	14.56 (14.15–14.97)	
Vigorous physical activity					< 0.001^†^
Everyday	14,991	27.60 (27.25–27.96)	24.57 (24.10–25.05)	30.89 (30.36–31.42)	
More than once a week	4417	7.59 (7.38–7.81)	5.25 (5.00–5.50)	10.13 (9.79–10.48)	
Once a week	2293	3.86 (3.71–4.02)	3.46 (3.27–3.67)	4.30 (4.07–4.54)	
1–3 times a month	3135	5.52 (5.34–5.71)	4.74 (4.51–4.98)	6.37 (6.09–6.66)	
Hardly ever or never	35,679	55.42 (55.02–55.81)	61.98 (61.44–62.52)	48.32 (47.75–48.89)	
Number of chronic diseases					< 0.001^†^
0	13,958	23.29 (22.96–23.63)	18.09 (17.66–18.52)	28.93 (28.41–29.45)	
1	14,297	24.19 (23.85–24.53)	22.15 (21.69–22.61)	26.40 (25.90–26.91)	
2	12,398	20.60 (20.28–20.92)	21.70 (21.24–22.16)	19.41 (18.96–19.87)	
≥ 3	19,862	31.92 (31.55–32.29)	38.07 (37.53–38.61)	25.26 (24.76–25.76)	
Drinking status					< 0.001^†^
Never	50,435	82.58 (82.28–82.88)	85.04 (84.64–85.43)	79.93 (79.46–80.38)	
Current	5758	9.91 (9.67–10.15)	8.69 (8.38–9.00)	11.23 (10.87–11.60)	
Ever	4322	7.51 (7.30–7.72)	6.28 (6.01–6.55)	8.84 (8.52–9.17)	
Smoking status					< 0.001^†^
Never	50,279	80.94 (80.63–81.25)	84.89 (84.49–85.28)	76.68 (76.19–77.16)	
Current	7741	15.02 (14.74–15.31)	11.43 (11.08–11.79)	18.90 (18.45–19.35)	
Ever	2495	4.04 (3.88–4.20)	3.68 (3.48–3.90)	4.42 (4.19–4.67)	
Depression					< 0.001^†^
No	40,286	64.50 (64.12–64.88)	67.51 (66.99–68.02)	61.25 (60.69–61.81)	
Yes	20,229	35.50 (35.12–35.88)	32.49 (31.98–33.01)	38.75 (38.19–39.31)	
Marital status					0.008^†^
Married or partnered	47,302	79.17 (78.85–79.49)	79.37 (78.91–79.81)	78.96 (78.49–79.43)	
Widowed	11,793	18.68 (18.37–19.00)	18.35 (17.93–18.79)	19.04 (18.59–19.49)	
Others	1420	2.14 (2.03–2.26)	2.28 (2.12–2.45)	2.00 (1.84–2.17)	
Living arrangement					< 0.001^†^
Co-residential living	58,576	96.83 (96.68–96.96)	97.62 (97.45–97.78)	95.97 (95.73–96.19)	
Separate living	1939	3.17 (3.04–3.32)	2.38 (2.22–2.55)	4.03 (3.81–4.27)	
Place of residence					< 0.001^†^
Urban	20,725	31.05 (30.68–31.42)	52.15 (51.60–52.70)	8.22 (7.91–8.55)	
Rural	39,790	68.95 (68.58–69.32)	47.85 (47.30–48.40)	91.78 (91.45–92.09)	
Economic status					< 0.001^†^
Low	20,593	36.99 (36.61–37.38)	24.75 (24.28–25.23)	50.23 (49.65–50.80)	
Middle	20,255	33.33 (32.96–33.71)	35.10 (34.58–35.63)	31.42 (30.89–31.96)	
High	19,667	29.68 (29.31–30.04)	40.14 (39.60–40.69)	18.35 (17.91–18.80)	
Religion					< 0.001^†^
Hindu	44,524	81.26 (80.95–81.57)	79.92 (79.47–80.36)	82.71 (82.27- 83.14)	
Muslim	7135	12.03 (11.78–12.29)	12.09 (11.74–12.46)	11.97 (11.60–12.34)	
Christian	6036	3.10 (2.97–3.24)	3.44 (3.25–3.65)	2.73 (2.55–2.93)	
Others	2820	3.61 (3.46–3.76)	4.54 (4.32–4.78)	2.60 (2.42–2.79)	
Caste					< 0.001^†^
Scheduled caste	10,265	19.90 (19.59–20.22)	16.23 (15.83–16.65)	23.87 (23.39–24.37)	
Scheduled tribal	10,745	8.65 (8.43–8.88)	4.14 (3.93–4.37)	13.52 (13.13–13.92)	
Other backward class	23,125	45.04 (44.65–45.44)	46.92 (46.37–47.47)	43.01 (42.44–43.58)	
None of the above	16,380	26.41 (26.06–26.76)	32.70 (32.18–33.22)	19.60 (19.15–20.06)	

Data were presented as weighted percentages or means (95% confidence intervals)

*BMI* Body mass index

^*^For continuous variable, *p* value was calculated by Kruskal Wallis H test

^†^For categorical variables, *p* value was calculated by chi-square test

### Fuel use and insomnia symptoms

Table 2 presents the associations of fuel use with insomnia symptoms and each of the symptoms. In the unadjusted model, polluted fuel use was significantly related to the report of insomnia symptoms (OR 1.23; 95%CI 1.17–1.30). After adjusting for demographic and socioeconomic characteristics (model 1), the relationship remained significant (OR 1.17; 95%CI 1.10–1.25). Further adjustment for biological covariates in model 2 did not alter this association (OR 1.17; 95%CI 1.10–1.25). Eventually, in the fully adjusted model 3, which additionally adjusted for environmental covariates, the association between polluted fuel use and insomnia symptoms persisted (OR 1.16; 95%CI 1.08–1.24, likelihood ratio test *p* < 0.05, and Hosmer-Lemeshow test *p* > 0.05). The links between different fuel types and each of the three kinds of insomnia symptoms, including DIS, DMS, and EMA, were also examined separately. In those fully adjusted models, consistent positive associations with polluted fuel use were observed for the three insomnia symptoms (OR 1.14; 95%CI 1.05–1.24 for DIS, OR 1.12; 95%CI 1.03–1.22 for DMS, and OR 1.15; 95%CI 1.06–1.25 for EMA in model 3).

**Table 2:** Relationship between fuel use and insomnia symptoms

Fuel use	OR (95% CI)			
	Unadjusted model	Model 1	Model 2	Model 3
Clean fuel	Reference	Reference	Reference	Reference
Polluted fuel				
Insomnia symptoms	1.23 (1.17, 1.30)	1.17 (1.10, 1.25)	1.17 (1.10, 1.25)	1.16 (1.08, 1.24)
Difficulty in initiating sleep (DIS)	1.15 (1.07, 1.23)	1.15 (1.07, 1.25)	1.16 (1.07, 1.25)	1.14 (1.05, 1.24)
Difficulty in maintaining sleep (DMS)	1.17 (1.09, 1.25)	1.14 (1.05, 1.23)	1.13 (1.05, 1.23)	1.12 (1.03, 1.22)
Early morning awakening (EMA)	1.24 (1.16, 1.33)	1.15 (1.07, 1.25)	1.16 (1.07, 1.26)	1.15 (1.06, 1.25)

Unadjusted model: no covariates were adjusted

Model 1 adjusted for: age categories, sex, level of education, work status, marital status, living arrangement, place of residence, economic status, religion, caste

Model 2 adjusted for: model 1 plus body mass index (BMI), vigorous physical activity, number of chronic diseases, drinking status, smoking status, depression

Model 3 adjusted for: model 2 plus other indoor pollution, indicators of poor housing quality

*OR* Odds ratio, *95% CI* 95% Confidence interval

### Subgroup analysis

**Fig. 2:**
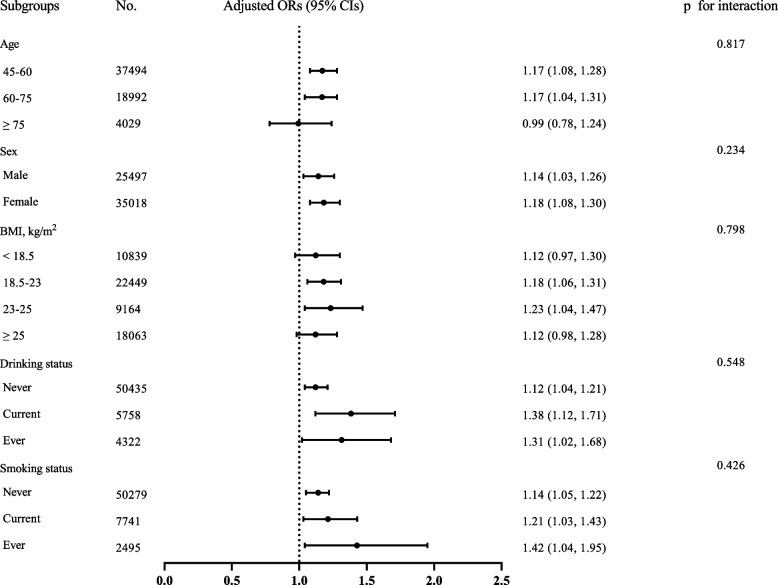
Subgroup analysis of the association between fuel use and insomnia symptoms. OR: odds ratio; 95% CI: 95% Confidence interval; BMI, body mass index. Model 3 adjusted for: age categories, sex, level of education, work status, marital status, religion, place of residence, living arrangement, economic status, caste, body mass index (BMI), vigorous physical activity, number of chronic diseases, drinking status, smoking status, depression, other indoor pollution, and indicators of poor housing quality except for the subgroup variable

To further assess the relationship between different fuel types and insomnia symptoms, we conducted heterogeneity analyses in different subgroups, the results of which are presented in Fig. 2. Our sample was stratified by age categories, sex, BMI, drinking status, and smoking status. In general, the ORs of each subgroup were in accordance with the main association results, suggesting a positive link between polluted fuel use and insomnia symptoms. After full adjustment, no significant interaction effect was observed in subgroup analyses by age categories, sex, BMI, drinking, or smoking status for the associations between fuel use and insomnia symptoms (*p* = 0.817, 0.234, 0.798, 0.548, 0.426, respectively).

## Discussion

Nearly half of (weight percentage 48.04%) participants relied on polluted household fuels to meet their domestic needs. Participants using polluted fuels tended to be older, lower education level, employed, unmarried, living alone, residing in rural regions, lower economic status, Hindu, the scheduled castes and scheduled tribes, BMI < 23 kg/m^2^, regular physical activity, current smoker, and drinker, lower exposure to other indoor pollution, and poorer housing quality. Approximately one in every ten middle-aged and elderly Indians (weight percentage 9.90%) experienced at least one insomnia symptom ≥ 5 times per week. Considering India’s substantial population base, insomnia poses a considerable economic burden on the country’s healthcare system. Moreover, functional and cognitive decline related to insomnia contributes to a decrease in the labor force [37], which would further strain the Indian economy that is already grappling with the additional healthcare expenses. Polluted fuel use was found to be associated with self-reported insomnia symptoms among middle-aged and elderly Indians, highlighting the need for public health initiatives geared toward addressing household environments, with particular attention to cooking fuels, with an aim to help alleviate the burden of insomnia symptoms on the population. Intriguingly, although the ORs of the associations between different fuel types and insomnia symptoms varied across subgroups, the *p*-value for interaction was found to be greater than 0.05, indicting no significant interactions for the associations between different fuel types and insomnia symptoms were observed for analyses stratified by age, sex, BMI, drinking, or smoking status. Consequently, our results suggest a consistent and stable association of fuel use with insomnia symptoms across different age categories, sexes, BMIs, alcohol consumption patterns, and smoking statuses.

Publications on air pollution and sleep disruption have focused on AAP [15–19]. A few studies conducted in China have explored the link of fuel use with sleep health. Chen et al. carried out a case-control study in Hainan, China of 1616 participants and found that exposure to polluted fuel use was linked to poor sleep quality at the oldest-old age (≥ 80 years), with cooking ventilation in those kitchens possibly attenuating the relationship in that the increased airflow might offset the detrimental effect of the incomplete combustion of polluted fuels [25]. Chair et al. performed a large cross-sectional analysis of 283,170 adults using the China Kadoorie Biobank Study, reporting that household polluted fuels were correlated with sleep disturbance [22]. Yu et al. declared that continuous usage of polluted fuels was related to shorter sleep time among individuals aged over 45 years (*n* = 8668) employing China Health and Retirement Survey [26]. Liao et al. in a study enrolling 28,135 rural participants in Henan, China, observed associations of poor sleep with combined fuel types and cooking time, which were modified by either natural (open windows or doors) or mechanical (exhaust hoods/fans) kitchen ventilation [23]. Wei et al. performed a cross-sectional study of 2197 employees in a Machinery Company in Liuzhou, China, and showed that there was an exposure-dose association between oil fumes and poor sleep [24]. Although our main findings regarding the association between polluted fuel and insomnia symptoms align with those of the Chinese studies, there are still variations that can be attributed to cultural factors. For example, we observe a significant urban–rural conundrum in India, as documented in existing literature [38], in terms of the utilization of polluted fuels. Our study indicates that approximately 91.78% (weighted 95%CI 91.45–92.09) of Indians using polluted fuels reside in rural areas, while the proportion in China is approximately 71.5% [26]. Despite the Indian government’s cooking energy program - *Pradhan Mantri Ujjwala Yojana* (PMUY) scheme – which has been launched to increase India’s LPG adoption [39], our findings suggest that a considerable number of rural Indian households still rely on traditional polluted fuels, implying that addressing cooking energy in rural areas should be a primary focus for the Indian government. Additionally, our study reveals demographic differences in fuel utilization that also reflect India’s unique cultural characteristics, such as religion and caste. Specifically, participants using polluted fuels were predominantly Hindu and belonged to the scheduled castes and scheduled tribes, a finding that has not been previously reported.

Existing literature suggests the possible biochemical mechanisms underlying the link of polluted fuel use with insomnia. Disturbance of sleep regulation in the central nervous system (CNS) could be a potential explanation. Pollutants from polluted fuel smoke have been related to decreased volume of the prefrontal cortex [40], cerebrovascular damage [41], and neurotoxicity [42]. Such CNS damage might affect cerebral areas involved in sleep and ventilatory regulation and consequently affect sleep quality. Exposure to air pollutants sourced from indoor polluted fuel use, such as PM_2.5_, has been associated with lower levels of serotonin [43], which interacts with other neurotransmitters to modulate sleep and circadian rhythms, leading to alterations in sleep patterns [44]. Inhaled air pollutant particles and diverse environmental toxicants absorbed by such particles might be transferred into the brain via altered blood–brain barrier and translocation of nanoparticles, initiating astrogliosis, microglial infiltration, and neuronal loss and causing inflammation in the brain [45, 46]. Circulating inflammatory cytokines might also cross from the periphery to the brain indirectly by systemic inflammation and neuroimmune cross-talk [47], ultimately triggering a cascade of neuroinflammation that affects the brain’s function and therefore influences sleep. Finally, both human and animal studies have proposed that pollutants from polluted fuel smoke are linked to increased expression of pathological markers of neurodegenerative diseases, e.g., alpha-synuclein, beta-amyloid and hyperphosphorylated tau [48], and may thus linked with the progression of neurodegenerative diseases. Besides, the mechanisms linking neurodegeneration to circadian dysfunction and impaired sleep are elucidated [49] and suggested the progressive nature of sleep disruption throughout the course of neurodegeneration [50]. Therefore, it is speculated that the potential neurodegenerative effects of pollutants from HAP might be associated with the process of insomnia.

In addition to the CNS, other systems have also been found to be involved in the relationship of polluted fuel use with insomnia. One plausible mechanism might be the links between polluted fuel use, respiratory disease, and insomnia. Inhaled particulates from HAP might deposit in the airway, causing oxidative stress, subsequently stimulating alveolar macrophages (AMs) and damaging alveolar epithelium [51]. Besides, AMs filled with pollutant carbon particles are linked to poor antibacterial ability, thereby contributing to an increased risk of infection and immunodeficiency [52]. These pathological changes in the respiratory system are associated with airflow obstruction, contributing to apnea and hypoxia, and further impacting sleep [53].

Another possible link between polluted fuel use and insomnia symptoms is cardiovascular autonomic dysfunction. Recent studies have extended associational evidence of polluted fuel use with adverse cardiovascular effects through mediating pathways, including autonomic imbalance [54]. Meanwhile, sleep physiology might be influenced by impaired autonomic control of the cardiovascular system, which may result in an imbalance of sympathetic or parasympathetic modulation during the sleep-wake cycles, eventually perturbing sleep [55]. Finally, mental factors might mediate the association between polluted fuel use and insomnia. Pollutants sourced from polluted fuel use might be associated with multiple adverse mental health outcomes, for instance, depression, anxiety, bipolar disorder, or suicide [56]. These negative emotional impacts caused by HAP sourced from polluted fuel could in turn influence falling asleep, a possibility supported by studies reporting a reciprocal relationship between mental disorders and sleep quality [57].

### Limitations

Our study has limitations. The cross-sectional nature of baseline LASI data cannot guarantee causality. However, future follow-up studies of LASI may provide additional support for establishing longitudinal causality. Additionally, the self-reported data on insomnia symptoms and other health conditions, such as chronic diseases, may be subject to reporting errors, including recall bias, idiosyncratic interpretation, etc. besides, data selection of this study is restricted to variables collected in baseline LASI, data on cooking duration, kitchen ventilation, past fuel use, or stacking with other fuel types were not included in the LASI database, which may lead to under- or over-estimation of the results. We also could not study the pollutant concentration, exposure time, or lifelong effect due to the limitation of the data availability. Since exposure to polluted fuels is a crude proxy of HAP and there was no estimate of AAP exposure, measurement bias might exist. Finally, despite extensive adjustment for potential covariates, the influence of uncontrolled covariates cannot be ruled out.

### Strengths

However, our study has several strengths. To our knowledge, this is the first attempt to explore the link between polluted fuel use and insomnia symptoms using very recent data available nationally representative of the Indian population, which allows us to generalize our findings to the full middle-aged and elderly population of India. Moreover, our analyses adjusted for multiple potential covariates. Thus, the results are robust and conducive to policy formulation. Furthermore, our results indicate an association between polluted fuel use and insomnia symptoms, thus further suggesting the need for future longitudinal studies to explore potential toxicological and biological mechanisms that underlie it.

## Conclusions

Nearly half of the Indian population is regularly exposed to polluted fuel use, which is associated with insomnia symptoms among middle-aged and elderly Indians. Polluted fuel use remains a major public health concern in India, with domestic inequalities driven by unbalanced developments. The implementation of national indoor environmental protection policies should be accelerated and reoriented to rural residents. More efforts are needed to shift to cleaner fuels and cooking technologies and could be combined with information campaigns to raise awareness of rural households. Future prospective study is warranted to validate our findings, to explore underlying mechanisms, and to inform policy and practice.

## Data Availability

The datasets generated and/or analyzed during the current study are available in the [Gateway to Global Aging Data] repository, (https://lasi-india.org).

## Abbreviations

LASI: Longitudinal Aging Study in India
DIS: Difficulty in initiating sleep
DMS: Difficulty in maintaining sleep
EMA: Early morning awakening
BMI: Body mass index
LMICs: Low- and middle-income countries
PM_2.5_: Particulate matter 2.5
CO: Carbon monoxide
BC: Black carbon
HAP: Household air pollution
COPD: Chronic obstructive pulmonary disease
DALYs: Disability-adjusted life years
AAP: Ambient air pollution
LPG: Liquefied Petroleum Gas
SES: Socioeconomic status
WHO: World health organization
CES-D-10: 10-Item Center for Epidemiologic Studies Depression Scale
CIs: Confidence intervals
CNS: Central nervous system
AMs: Alveolar macrophages
